# How to train a self-driving vehicle: On the added value (or lack thereof) of curriculum learning and replay buffers

**DOI:** 10.3389/frai.2023.1098982

**Published:** 2023-01-25

**Authors:** Sara Mahmoud, Erik Billing, Henrik Svensson, Serge Thill

**Affiliations:** ^1^Interaction Lab, School of Informatics, University of Skövde, Skövde, Sweden; ^2^Donders Institute for Brain, Cognition, and Behaviour, Radboud University, Nijmegen, Netherlands

**Keywords:** data generation, curriculum learning, cognitive-inspired learning, reinforcement learning, replay buffer, self-driving cars

## Abstract

Learning from only real-world collected data can be unrealistic and time consuming in many scenario. One alternative is to use synthetic data as learning environments to learn rare situations and replay buffers to speed up the learning. In this work, we examine the hypothesis of how the creation of the environment affects the training of reinforcement learning agent through auto-generated environment mechanisms. We take the autonomous vehicle as an application. We compare the effect of two approaches to generate training data for artificial cognitive agents. We consider the added value of curriculum learning—just as in human learning—as a way to structure novel training data that the agent has not seen before as well as that of using a replay buffer to train further on data the agent has seen before. In other words, the focus of this paper is on characteristics of the training data rather than on learning algorithms. We therefore use two tasks that are commonly trained early on in autonomous vehicle research: lane keeping and pedestrian avoidance. Our main results show that curriculum learning indeed offers an additional benefit over a vanilla reinforcement learning approach (using Deep-Q Learning), but the replay buffer actually has a detrimental effect in most (but not all) combinations of data generation approaches we considered here. The benefit of curriculum learning does depend on the existence of a well-defined difficulty metric with which various training scenarios can be ordered. In the lane-keeping task, we can define it as a function of the curvature of the road, in which the steeper and more occurring curves on the road, the more difficult it gets. Defining such a difficulty metric in other scenarios is not always trivial. In general, the results of this paper emphasize both the importance of considering data characterization, such as curriculum learning, and the importance of defining an appropriate metric for the task.

## 1. Introduction

Autonomous vehicles such as self-driving cars operate in the real world, which means that the driving agent has to handle a wide range of different tasks and scenarios such as lane following, negotiating traffic and intersections, communicating (explicitly or implicitly) with pedestrians, etc. Since all these situations are safety-critical, it is also critical that the autonomous vehicle is capable of handling them in all foreseen and unforeseen guises. For example, when negotiating intersections, it must be able to handle any type of intersection, various traffic loads, the potential presence of pedestrians, and other vulnerable road users, and so on.

As with all such real-world situations, it is not feasible (nor desirable to attempt) to fully and completely anticipate all possible situations that an autonomous vehicle might possibly encounter. Therefore, fully hard-coded solutions are simply impractical (Da Lio et al., 2017) and a significant portion of the research on autonomous vehicle control focuses on machine learning approaches, such as deep learning, to design a controller that can learn the appropriate skills. Deep learning, generally speaking, has been hugely successful in many tasks that are relevant for autonomous vehicles, most famously image processing (Geiger et al., 2013; Wali et al., 2015; Grigorescu et al., 2020). End-to-end deep networks are also successful at learning how to map sensory inputs onto motor outputs for the control of simple agents (Bojarski et al., 2016; Chen and Huang, 2017; Haavaldsen et al., 2019). These advances are possible because the innovations in deep neural networks (compared to earlier approaches) increased their ability to extract relevant data in the training set. The flip side of the coin is, however, that this places a large onus on the training set used: while we can be relatively confident that a modern deep learning algorithm will make close to optimal use of the training set, it cannot go beyond what the quality of the training set affords. One way this is dealt with traditionally is by having extremely large data sets (given that the available computing power is now at a point where their processing is feasible). However, for many real-world applications, collecting such data sets is usually not practical and often not possible even in principle. When it comes to autonomous vehicles, part of the reason is that critical situations are also rare: no amount of driving in sunny conditions can result in data on how to handle a spot of black ice in a corner at night.

While it is therefore possible to train controllers that do reasonably well in most common situations (the state-of-the-art of which can be observed at any time by evaluating the performance of commercial products), dealing with rare events that are both unlikely to be encountered often enough to be relevant in training data and critical in terms of safety remains a challenge. In robotics, attempts exist to address this challenge through reinforcement learning (RL), where the agent is given feedback on the quality of the actions taken and thus able to adapt. Rare situations can thus be trained explicitly when the agent encounters them. At the same time, it is clear that this cannot take place in the real world given the consequences of a poor decision on behalf of the controller (Zhao et al., 2020). There is thus a significant interest in training controllers for autonomous vehicles in simulation with auto-generated scenarios. Such scenarios can be entirely new situations or variations of actually encountered rare events to increase robustness (Da Lio et al., 2017). For example, initial training in tasks like lane-keeping could involve entirely new situations (if the vehicle encounters certain curvatures for the first time). A rare event, meanwhile, could concern a pedestrian crossing the street unexpectedly, where variations on speed of the pedestrian, location, degree of occlusion through other objects, and so on, could then be simulated.

An important distinction between deep learning and reinforcement learning concerns how the training phase should be structured given the mechanisms by which the agent actually learns in each. Deep learning is largely supervised and focuses on learning correct associations between inputs and outputs by providing a training set that contains both the input and the expected output for that input. For that reason, it is common to randomize the elements in the training set so that the algorithm can generalize over the entire set. In reinforcement learning, the agent learns through interactions with the world. Therefore, it is important to structure these interactions appropriately during training. For example, a fully untrained model might struggle to learn how to deal with particularly complicated situations, while a model that has mastered the basics can fare better (Kulkarni et al., 2016). Another issue is catastrophic forgetting (Parisi et al., 2019): while the vehicle needs to learn from novel situations, it should not forget how to handle those it has previously encountered. Overall, how to structure training using reinforcement learning so that skills can be learned accumulatively and appropriately is a challenging problem (Berseth et al., 2018).

At the same, humans clearly demonstrate an ability to learn in such a manner themselves. For humans, however, development and learning are rarely random. More often, it is a consequence of internal development and change and external guiding forces working in tandem. A simple example can be observed in formal education: most academic programs are structured according to a curriculum such that students learn more simple or basic concepts first before moving on to more complex concepts (Hacohen and Weinshall, 2019). In reinforcement learning, the approaches that take inspiration from this are therefore called curriculum learning (Narvekar et al., 2022). Specifically, curriculum learning suggests that it is beneficial to structure the exposure to learning examples, often progressively proceeding from initially simple tasks to increasingly complex ones (Elman, 1993; Bengio et al., 2009; Krueger and Dayan, 2009; Hacohen and Weinshall, 2019).

In this paper, we are interested in two aspects when it comes to the use of curriculum learning for autonomous vehicles. First, curriculum learning assumes that it is possible to structure training scenarios from “simple” to “complex.” This implies the existence of some difficulty metric that can be used to rank scenarios. However, in driving scenarios, this may not always be trivial to define. In a lane-keeping task, which is typically one of the first tasks that any autonomous vehicle controller needs to learn (Bojarski et al., 2016; Santana and Hotz, 2016; Ha and Schmidhuber, 2018; Bae et al., 2021), such a metric could be defined based on the curvature of the road, number of corners, and so on, since these are the aspects that interfere with keeping the vehicle in the center of a lane. In a pedestrian avoidance task, the difficulty for scenarios can be defined by the trajectory of the pedestrian and assessments of whether the pedestrian is, for example, careful or reckless; however, even if such metrics can be found, it is much less clear how the difficulty of one scenario to the other could be quantified. The second aspect we are interested in is the actual benefit of structured data in general and of curriculum learning in particular. In other words, we are interested in the core assumption hat there is a benefit to structure in the training set, as there is to human learning.

We use reinforcement learning to train autonomous vehicles on two tasks, lane keeping, and pedestrian avoidance. These are chosen because they are standard tasks on which any controller is trained and because one of them has a clearly defined difficulty metric, while the other does not. As the main focus is on the training data, we implement multiple ways to generate synthetic training scenarios for autonomous vehicles (where we use the word “synthetic” to denote that these scenarios are not hard coded or explicitly designed by the programmer but are specified and generated by an auto-generation mechanism), in particular with and without a curriculum structure.

For the lane keeping task, we find that structured learning environments improve the quality of the learned controller only if it is not used in combination with a replay buffer (a technique in reinforcement learning that allows the reuse of previously encountered scenarios when training on a novel one). For pedestrian avoidance task, we do find that a non-structured scenario generation approach is already sufficient for the controller to perform well-enough (i.e., avoiding the pedestrian in all test cases).

Overall, the contributions are therefore two-fold: first, we demonstrate that curriculum learning does not necessarily provide an added value, so the inability to define an appropriate difficulty metric is not always a relevant hurdle. Second, however, when it does provide a benefit, this depends on other aspects of the algorithm: in our case, additionally using a replay buffer (for which there can be good reasons) had a detrimental effect in our structured learning scenarios.

In the remainder of the paper, we first detail our methods for generating synthetic training scenarios and our implementation of reinforcement learning. We then describe how both lane-keep and pedestrian avoidance tasks are designed. This is followed by the main results: for the lane keeping task, we demonstrate performance on a baseline as well as on different synthetically generated training scenarios. For pedestrian avoidance, we only present results on one training regime, as there is no clear quantitative metric for how to order the scenarios in terms of difficulty for the autonomous vehicle, making curriculum learning difficult. We conclude with a discussion relating these results to the wider literature and suggestions for future work.

## 2. Background

### 2.1. Types of synthetic training data

As discussed in the introduction, we focus on different mechanisms to structure the sequence of synthetic training scenarios that the vehicle encounters. In principle, this implies that we will generate novel scenarios in simulation and either sort them in order of some difficulty metric, or not. This corresponds to purely off-line training of a vehicle in simulation before it is used on a road.

However, it is also possible to simply learn from past experience rather than to generate such novel scenarios. This would correspond to a vehicle that actually has encountered a certain situation on the road and is now “replaying” it to learn and improve its behavior in this situation. In the literature, this is typically referred to as, and implemented using, a replay buffer (Lin, 1993; Mnih et al., 2013, 2015).

Naturally, it is also possible to combine the two. In this paper, as detailed below, we will explore the performance of the various combinations of scenario generation (none, random order, ordered by difficulty) and use of a replay buffer (not used used).

### 2.2. Synthetic scenario generation

There are different approaches to generate complete scenarios for a learning agent without hard-coding them. One approach is to use a Generative Adversarial Network (GAN) (Goodfellow et al., 2014), in which a network learns to generate new unseen data from a given data set. GANs have previously been used to train a neural network to generate video scenarios similar to the collected real-world video data. These scenarios were then validated by running a RL driving agent through them (Santana and Hotz, 2016), and initial results demonstrated that the synthetic data were close to the real.

Ha and Schmidhuber (2018) used a similar technique in an Acari driving car game. Their work illustrates the use of internal models in a deep learning setting. First, the car explores the environment to learn the model of the world. Next, it learns to generate new scenarios in the form of a sequence of images similar to those explored, albeit with slight differences that the car has not experienced before. The agent then attempts to learn (through RL) to drive in these new scenarios. In evaluation, the agent performed better when trained in the synthetic (unseen) environment before being injected back into the real environment. This illustrates that agents might benefit from unseen examples, similar to how humans gain a behavioral advantage from re-enacting hypothetical future situations (Revonsuo, 2000; Svensson et al., 2013; Billing et al., 2016; Gershman and Daw, 2017; Windridge et al., 2020).

However, one potential drawback of GAN lies in their stochastic nature. It is not trivial to retain control over the exact generation process or over what features are to be emphasized. To address this, scenarios can also be generated using a physics simulation engine. Such a simulation builds an environment from features specified, for example, through an API (Application Programming Interface). This allows for auto-generating novel scenarios while maintaining designer-imposed constraints. Such an approach is regularly used in robotics, and although the scenarios are often hard-coded in the literature (e.g., Gu et al., 2017), they do not have to be.

Curriculum learning, however, also considers the temporal aspect of the training phase, suggesting that faster training and higher performance can be achieved if the learning material is presented in an appropriate order. For example, ordering episodes in difficulty would result in a sequence of episodes [*Ep*_1_, *Ep*_2_, *Ep*_3_, *Ep*_4_...*Ep*_*m*_] in which, for each episode *Ep*_*k*_, episode *Ep*_*k*+*n*_ is more difficult than episode *Ep*_*k*_, where *m* is the number of episodes and *n* is a positive integer. Thus, curriculum criteria as sequencing metric is required to specify that episode *Ep*_*k*+*n*_ is more difficult than episode *Ep*_*k*_.

One aspect to consider is how and when to create training episodes. One possibility is to generate them all prior to learning. For example, Anzalone et al. (2022) used the ready made environments in CARLA to train a curriculum RL on all environments. Another alternative is to adapt the difficulty of episode creation based on the current performance of the agent (Narvekar et al., 2017). As we are interested in the effects of ordering episodes by difficulty, we generate them at the start with this criterion in mind and as such, the order is not determined during training in function of some aspect of the learning agent.

Another aspect is to determine when the agent moves from one episode to the next. The agent may repeatedly attempt some specific episode until performance plateaus (measured, e.g., by network error or accumulated rewards). However, recent research has found this to be unnecessary and that the agent can simply move to the next episode upon completion (Narvekar and Stone, 2019).

This incremental structure in the sequence of episodes assumes that difficulty is measured by certain metrics that can be used to compare episodes. Therefore, that some metric *C* exists such that |*C*_*E*_*p*__*a*__| < |*C*_*E*_*p*__*b*__|. For example, Camargo and Sáenz (2021) proposed curriculum learning for game engine. The agent plays the same game (e.g., soccer) but the game parameters such as the opponent speed and reward function varies between episodes. In the lane keeping scenario, this is measured by the degree of curvature in the road segments as well as the frequency of their occurrence in the same road length. In the pedestrian avoidance scenario, however, there are many candidate variables that might contribute to such a metric, including the trajectory of the pedestrian, the speed at which they are moving, and more. Rare and unusual events cannot be excluded; for example, the pedestrian may start to cross a road but then reverse direction. Although the sequence of episodes may includes such, or other, varieties, there is no unambiguous way to measure and order the difficulty of these episodes. This is often an issue, and in these cases, an expert human can order episodes manually, relying on their domain knowledge rather than some simple metric. This is referred to as human-in-the-loop curriculum generation (Narvekar et al., 2022). Here, the episodes used in the pedestrian avoidance task are essentially randomly ordered. Since this was sufficient to solve the task, as detailed below, we did not investigate specific orderings further. One could however consider stable pedestrian trajectories to be easier than trajectories with frequent direction changes. Similarly, a pedestrian who is partially or wholly occluded before crossing the road would be part of a more difficult episode than a pedestrian who remains visible at all times.

### 2.3. Replay buffer

Reinforcement learning is usually a sequential process where the agent learns experiences in the order it encounters them, but the addition of a replay buffer allows the agent to preserve older experiences. The agent accumulates the previous experiences of the state and action in a buffered memory and learns them to improve the policy. One of the earliest approaches that use a replay buffer (also known as experience replay) in reinforcement learning was the Dyna architecture (Sutton, 1991). Lin (1993) suggested that it might be useful to introduce experience replay to a simulated mobile robot to speed up the credit assignment making the agent more likely to remember what it had learned before. Later work with deep reinforcement learning has demonstrated the utility of replay buffers for deep reinforcement learning in several tasks (Mnih et al., 2013, 2015; Lillicrap et al., 2015).

A replay buffer is not about creating novel scenarios, but about remembering previous experiences to better learn from them. In the case of a reinforcement learning agent, the learning agent accumulates (in the replay buffer) previous experiences (or transitions) in the form of a quadruple (*s*_*t*_, *a*_*t*_, *r*_*t*+1_, *s*_*t*+1_), which can be interpreted as the execution of an action *a*_*t*_ in a state *s*_*t*_ results in a new state *s*_*t*+1_ and rewards *r*_*t*+1_ (Lin, 1993; Vanseijen and Sutton, 2015; Zhang and Sutton, 2017). It may then reuse them to access parts of previous scenarios without explicitly perceiving the transition from the sensory data (e.g., Gershman and Daw, 2017).

Although typical replay buffers are based on stochastic retrieval of experiences from a buffered memory, recent efforts have attempted to improve this mechanism (Horgan et al., 2018). Schaul et al. (2016) for example, proposed a prioritized experience replay, in which the latest experiences receive higher priority to be repeated than the older ones. One of the drawbacks of the prioritized experience replay is that it increases the complexity of the algorithm from *O*(1) to *O*(*logN*) (Zhang and Sutton, 2017). Instead of storing the experience weight for each entry and restructure them for retrieval, Kim and Choi (2018) used two neural networks; one as the main network and another as a secondary network. The secondary network, referred as ScreenerNet, learns to predict the weight of the experience based on the error of the main network.

## 3. Methods

### 3.1. Experimental setup and tasks

Our basic experimental setup is a simulated autonomous vehicle that learns to drive on test roads. It can do so purely based on performance on a training road, or using, in addition, various additional synthetically generated scenarios. Therefore, not using any synthetic training data implements a vanilla RL agent that serves as a baseline. We also investigate two different tasks, low-level control of lane-keeping and higher-level decision-making for pedestrian avoidance task.

### 3.2. System architecture

**Figure 1:**
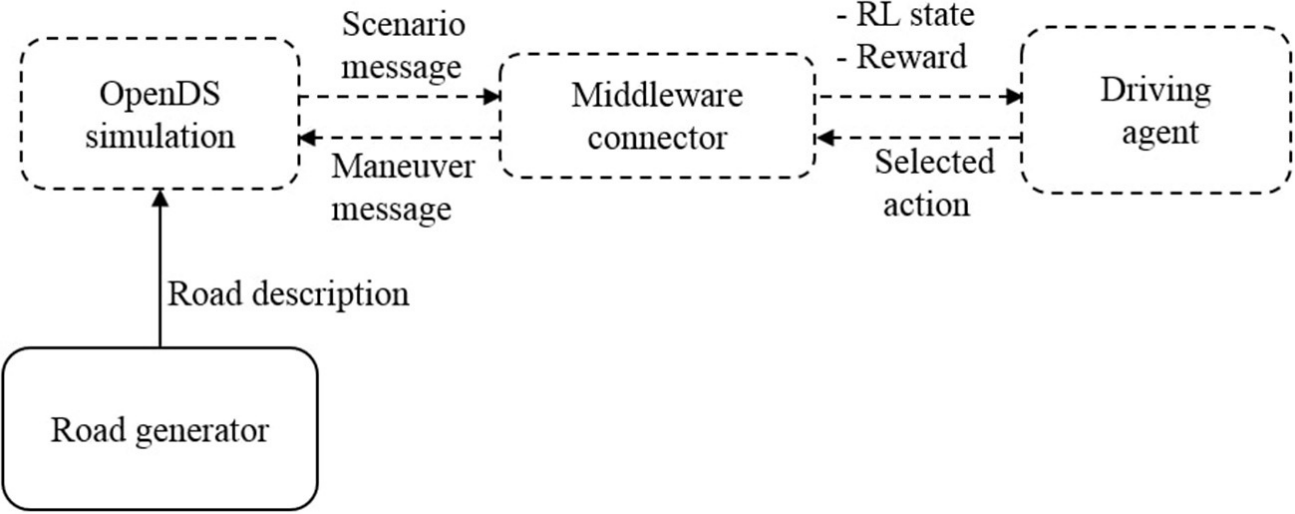
Episodic generator system architecture for self-driving car on OpenDS simulation.

The system architecture consists of four main components: (1) OpenDs, the physical simulation, in which the training and testing are executed, (2) a middleman connector that converts the simulation into a RL environment, (3) a learning driving agent, and (4) a road generator for creating novel scenarios (see Figure 1).

**Figure 2:**
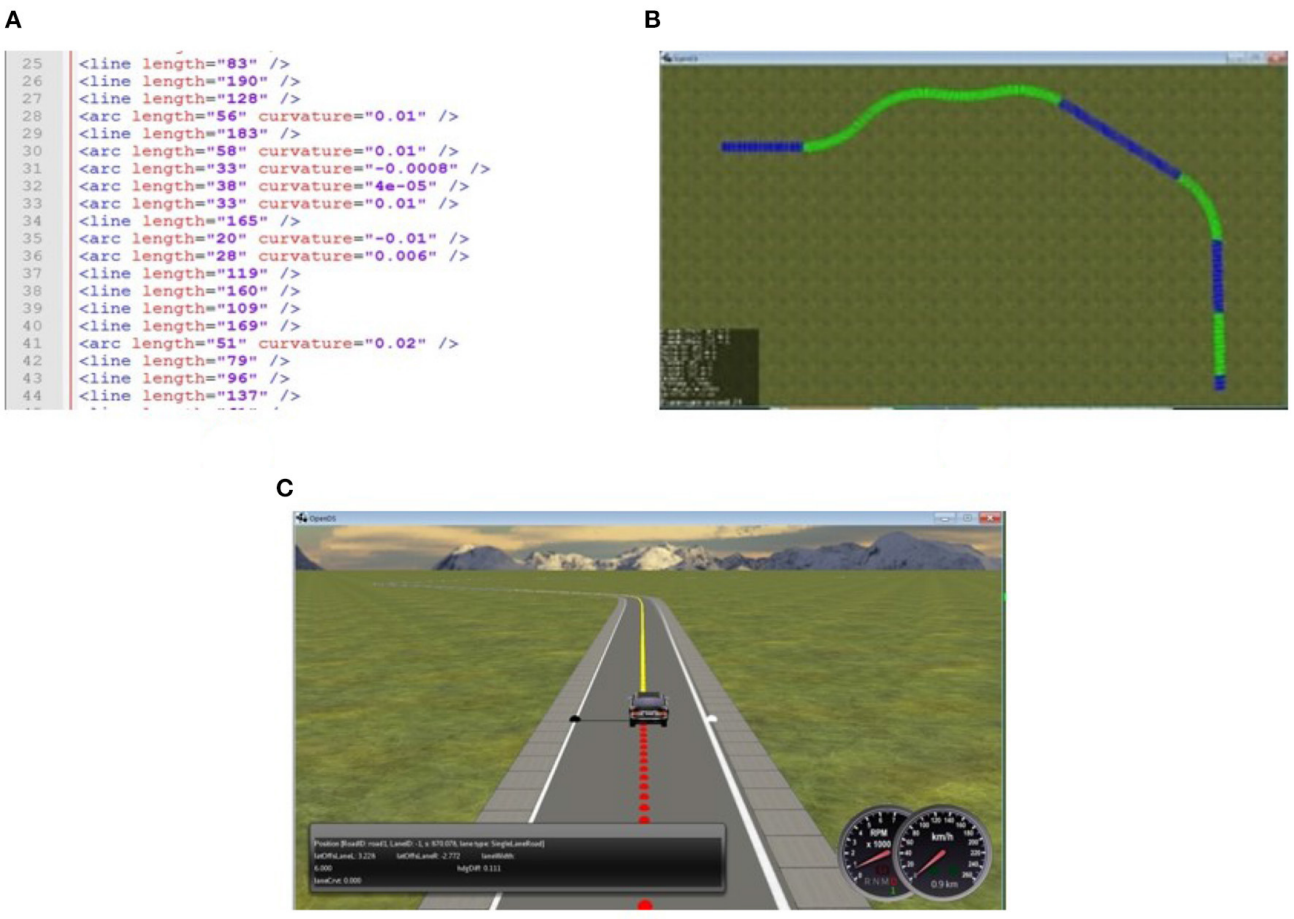
From road description in XML to rendered road in OpenDS. **(A)** XML road description sample. **(B)** Bird view of a 3D rendered road in Opens. **(C)** 3D rendered road.

*Opens Simulation*^1^ is an open source driving simulator with a multi-threaded physics engine, that allows in particular mesh-accurate collision shapes and enables the application of basic forces such as acceleration, friction, torque, gravity, and centrifugal forces during simulation. The main motivation for using OpenDS is that it provides a manner of creating episodic generation using APIs that is viable with respect to complexity. The API uses Extensible Markup Language (XML) files that are both human-readable and machine-readable. This allows algorithmic generation of the environment specifications in the XML files (see Figure 2A) which can then be rendered into the 3D simulation environment (see Figure 2B).

The middleware connector is required to implement a RL loop since OpenDS is just a simulator. We use this middleware connector to calculate the reward function at each step. The connector also acts as a communication bridge between OpenDS and the driving agent to form a state message to the agent and an action message to the maneuver message to the simulation (using a UDP connection with 20 messages per second).

The driving agent in this paper uses Deep-Q-Learning as the RL algorithm. In Deep-Q-Learning, the calculation of the state-action value function (Equation 1), is independent of the policy (Sutton and Barto, 2018). In other words, the agent is able to calculate the value of the next state without calculating the policy. The policy (π) in DQL is to select the action that outputs the maximum *Q* value (Equation 2).


(1) $$\begin{array}{l} Q^{\pi }\left(s,a,\theta \right)=E\left\{r_{t+1}+\gamma \underset{a^{\prime }}{\max}Q^{\pi }\left(s_{s+1},a^{\prime },\theta_{i-1}\right)|s_{t}=s,a_{t}=a\right\} \end{array}$$

(2) $$\begin{array}{l} a_{t}=argmax_{a}Q\left(s_{t},a,\Theta \right) \end{array}$$

Where *r*_*t*_ is the reward received, *s*_*t*+1_ is the next state, *a*′ is the action that would be taken in the next state and Θ are the network's hyperparasite.

The network is trained to minimize the loss at each step [defined as the mean square error between the predicted *Q*(*s*_*i*_, *a*_*i*_) value and the actual value *y*_*i*_, Equation (3)] and the goal is to reduce the network loss for the weights Θ, calculated as in Equation (4).


(3) $$\begin{array}{l} y_{i}=\{\begin{array}{ll} r_{t}, & \text{terminal state}\\ r_{t}+\gamma max_{a^{\prime }}Q\left(s_{t+1},a,\theta_{i-1}\right), & \text{non terminal state} \end{array} \end{array}$$

(4) $$\begin{array}{l} L_{i}\left(\theta_{i}\right)={\text{𝔼}}_{s,a}\left[{\left(y_{i}-Q\left(s,a,\theta \right)\right)}^{2}\right] \end{array}$$

The neural network of the learning agent used in our experiments consists of four layers [4, 30, 60, 3]. The input layer has four neurons for the RL state values, followed by two fully connected hidden layers with sizes of 30 and 60, both with a Rectified Linear Unit (ReLU) activation function. Finally, the output layer has three neurons with a linear activation function for the three available actions. The RMSprop optimizer is used. The state is normalized to have values between 0 and 1. The value of the exploration rate (ϵ) defines the probability of selecting a stochastic or deterministic policy at each step and here starts with 1 for the first 1,000 steps and decreases by 10^−7^ at each step until it becomes steady at 0.2. Hyperparasite are summarized in Table 1. During our study, we made some modifications to the exploration rate of the traditional decay method. We allowed ϵ to increase by 0.25 when using the exploitation strategy, or following the current policy, does not lead to success in 100 episodes in a row only if ϵ ≤ 0.5. This allows the driving agent to explore even in the later stages of training.

**Table 1:** Experiment hyper-parameters for the learning in the three environment generation approaches.

**Hyper-parameter**	**Value**
State space	[LatOffsLaneL, SteerWhlAg, LaneCrvt, LaneHeading]
Action space	[0.05, 0, −0.05]
Network size	[4, 30, 60, 3]
Network Optimizer	RMSprop
Input layer activation function	Linear
Hidden layer activation	ReLU
Output layer activation	Linear
Min reward	0
Initial (ϵ)	1
Decay	10^−7^
Final ϵ	0.2
Discount factor (γ)	0.9
UDP frequency	20 message per second

During training: (1) the driving agent receives a representation of the road through the middleman connector as previously described, (2) selects the action from a set of available actions, (3) receives the calculated reward signal, and (4) updates the weights of the network. This repeats until it reaches a terminal state. The terminal state is, in the lane-keeping task, defined as either a successful completion of a road or driving outside the road boundaries and, in the pedestrian avoidance task, defined as a collision with the pedestrian. Algorithm 1 describes the default algorithm for the learning agent.

**Algorithm 1:**
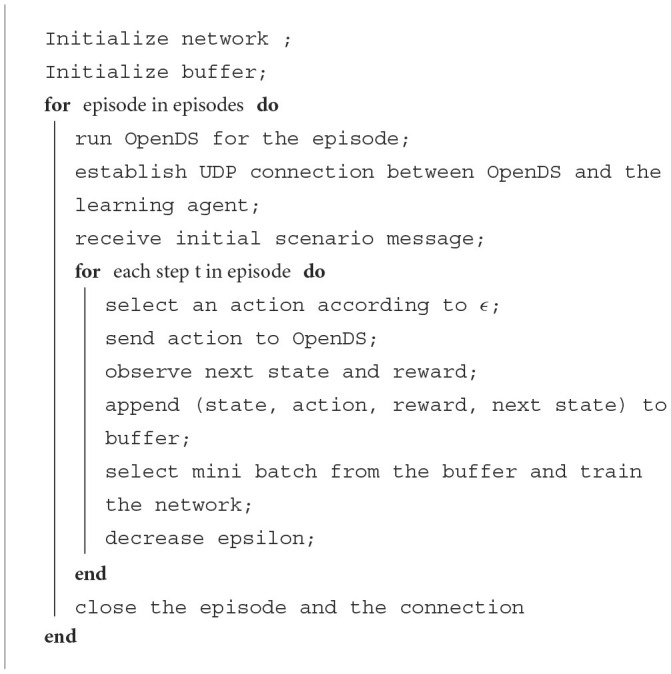
Learning algorithm for the driving agent.

Finally, the road generator automatically creates novel scenarios by generating road descriptions and storing them in XML format. These are then sent to OpenDS through APIS for road construction (see Figure 2). Figure 2A shows a sample of road descriptions produced by the road generator. Figure 2B illustrates the road map produced by the API in OpenDS. This runs as a learning environment, as shown in Figure 2C.

### 3.3. Lane keeping task

The first task is lane keeping, in which the goal of the driving agent is to learn the low-level wheel steering control for driving within the road lanes.

#### 3.3.1. Reward function

**Figure 3:**
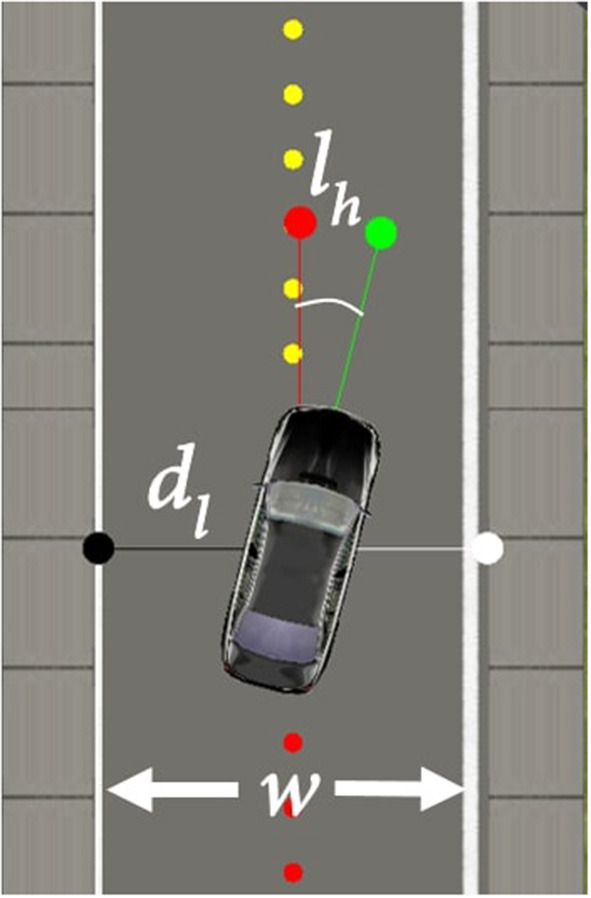
Lane keeping parameters.

The reward function, in this task, is based on the distance from the edge of the road (Equation 5) and the car heading angle (Equation 6), as shown in Figure 3. This results in the reward function (Equation 7), where *r*_*e*_ is the reward for the distance from the side of the road, *d*_*l*_ is the distance of the car from the left edge of the road in meters, *w* is the width of the lane in meters, *r*_*h*_ is the reward for the car heading, *l*_*h*_ is the angle between the car heading and the road heading in radius and *r*_*t*_ is the total reward. In simple terms, the function returns the highest reward when the car is in the middle of the lane and aligned with the direction of the road.


(5) $$\begin{array}{l} r_{e}=min\left(d_{l},w-d_{l}\right) \end{array}$$

(6) $$\begin{array}{l} r_{h}=2e^{\left(-15|l_{h}|\right)} \end{array}$$

(7) $$\begin{array}{l} r_{t}=r_{e}+r_{h} \end{array}$$

#### 3.3.2. Road generation

**Figure 4:**
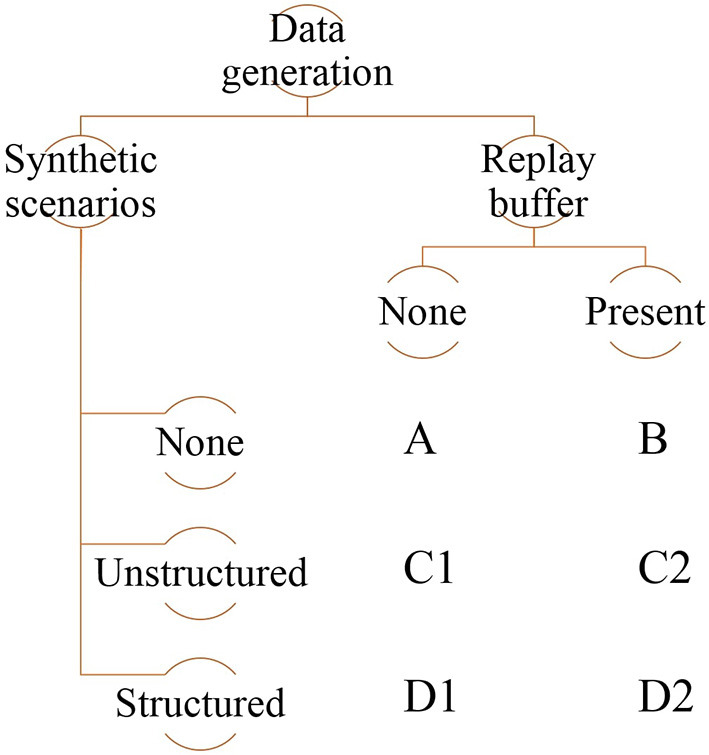
Summary of the different combinations of synthetic scenario generation and replay buffer use in this paper.

As detailed before, we distinguish between the creation of novel training scenarios and the reuse of past experience in training, without hard-coding either. The different combinations of ways to structure scenarios during learning and usage (or not) of a replay buffer are summarized in Figure 4. Notably, we also explore the option of using neither synthetic generation of novel training scenarios nor a replay buffer. In this case, the implementation reduces a standard Deep-Q-Learning implementation that serves as a baseline.

Our implementation of the replay buffer is based on the standard technique (Mnih et al., 2015). In terms of ways to generate novel scenarios, we primarily distinguish between unstructured and structured generation. In the latter, the sequence of scenarios the agent encounters is ordered by increasing difficulty (for the lane-keeping task, difficulty of a scenario is based on the curvature of the road generated for that scenario). An unstructured sequence, on the contrary, does not impose any such ordering and is therefore random.

For the lane keeping task, we implement all combinations of synthetic data generation and replay buffers (shown in Figure 4). The important aspects of road generation are the ratio of straight to curved segments and the geometries of the curves. All generated roads are single-lane with an approximate length of 550 m. Here, we describe these combinations in detail:

##### 3.3.2.1. Control condition [A]

**Figure 5:**
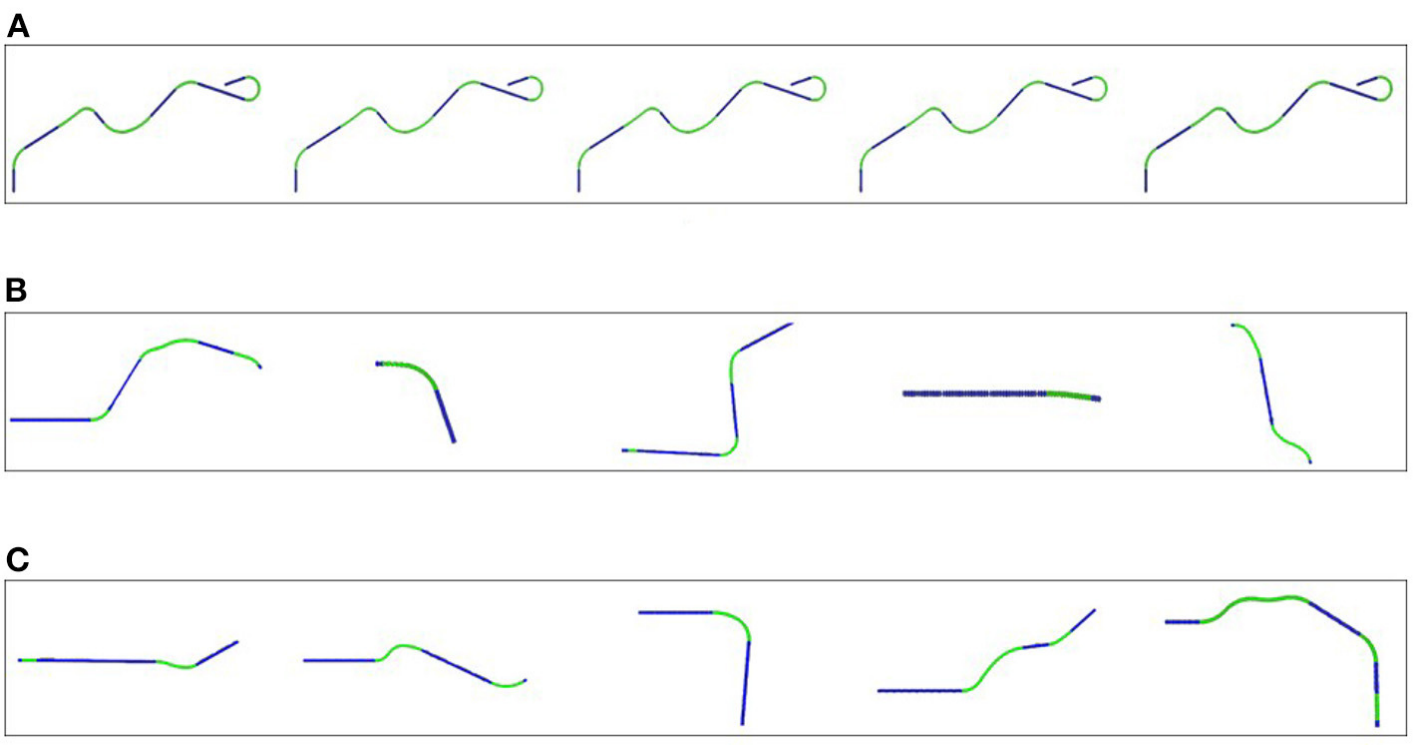
Examples of some roads the learning agent encounters in a sequence. **(A)** The same road is repeated throughout training. **(B)** A sequence of roads in a random order of difficulty used for training. **(C)** A sequence of roads ordered by increasing difficulty used for training.

As previously stated, the control condition serves as a baseline and implements a vanilla RL agent, similar to how simulations are typically used in other works (e.g., Sallab et al., 2017). A single randomly generated road is used (Figure 5A). This road is generated so that it contains a mix of straight and curved segments of various degree of curvature. Although it is a single road, it contains variations in curvature to produce, after training, an agent that is generally capable of keeping lanes. The agent repeatedly attempts to drive the entire length of the road and training ends when the agent successfully completes 100 consecutive drives.

##### 3.3.2.2. Replay buffer only [B]

In this condition a single road with random difficulty is used for training. The road scenario is the same as used for condition [A]. Condition [B] differs from condition [A] by adding a replay buffer. This buffer *D* is of size 1,000, which stores previous experiences *e*; so *D* = {*e*_1_, *e*_2_, *e*_3_, *e*_4_, … }. *e*_*i*_ itself is a tuple (*s*_*i*_, *a*_*i*_, *r*_*i*+1_, *s*_*i*+1_) that contains the current state, the selected action, the reward after executing the action and the next state, respectively. When the number of accumulated experiences exceeds the size of the replay buffer, an old experience is removed from the buffer, and the recent experience is added to the buffer. Instead of the standard approach of removing the oldest experience (Mnih et al., 2015), we set the agent to remove the oldest most practiced experience type. This is implemented by grouping experiences based on the curvature values and then ordering the groups by size. The oldest experience in the largest group is removed from the replay buffer, and the new experience is added to the replay buffer.

At each step, the agent randomly samples a mini batch of 36 experiences from the replay buffer and trains the network using a supervised learning gradient descent approach. The input data *X* for the network training is a 4 × 36 state matrix, while the output *Y* is the *Q* value for each action as a 3 × 36 matrix.

For the first 1,000 steps of training, the exploration rate (ϵ) is fixed at ϵ = 1, which means that the agent only follows a stochastic policy to explore the different actions.

##### 3.3.2.3. Unstructured synthetic data generation [C1]

In this condition the agent is trained in a number of scenarios, each containing a different road. The agent proceeds from one scenario to the next once it has successfully completed the current road. We generate 100 different roads in random order of difficulty before starting the training phase (see Figure 5B). The ratio of curves and straights is fixed to [40 curves : 60 straights] for all roads. For the different parts of the road, the length and the curvature were randomly chosen between 20 and 60 m and –0.015 and 0.015 *m*^−1^, respectively, summing up to a total length of 550 m.

##### 3.3.2.4. Unstructured synthetic data generation with a replay buffer [C2]

This condition is identical to condition [C1] in terms of road generation. It additionally uses a replay buffer implemented the same way as in condition [B].

##### 3.3.2.5. Structured synthetic data generation [D1]

In this condition structured data generation is used to generate the 100 roads in increasing order of difficulty (see Figure 5C): the first road is mostly straight, which then gradually increases the ratio of curves to straight segments to reach the ratio previously used [40 curves : 60 straight] after 40 roads. The curvature limits were set as (–0.007–0.007 *m*^−1^) for the first 40 roads, increasing to (–0.01–0.01 *m*^−1^) until road 80 and settled at (–0.015–0.015 *m*^−1^) for the last 20 roads.

##### 3.3.2.6. Structured synthetic data generation with a replay buffer [D2]

This condition is identical to condition [D1] in terms of road generation. It additionally uses a replay buffer implemented the same way as in condition [B].

### 3.4. Pedestrian avoidance through speed control task

**Figure 6:**
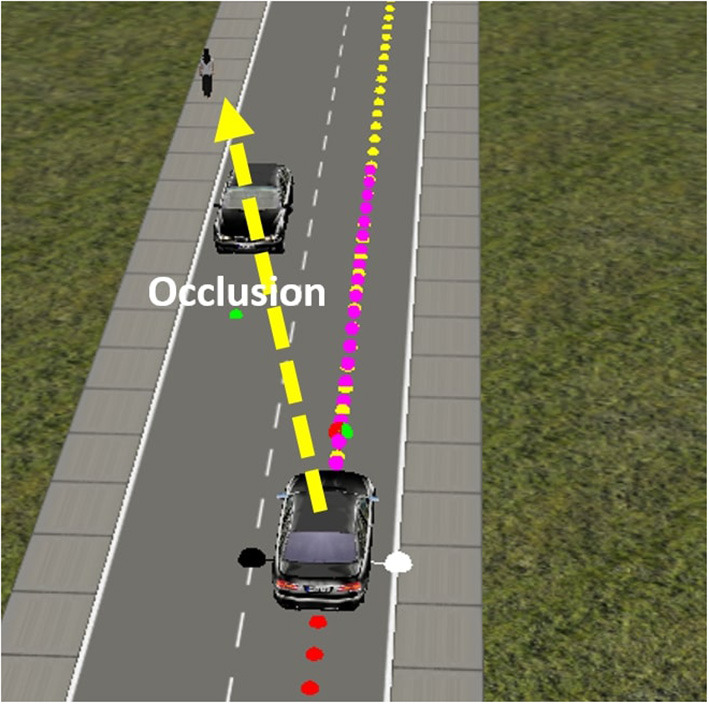
In the pedestrian avoidance task, the pedestrian may be occluded by a stationary vehicle at some point during the scenario.

The second task relates to higher-level decision making. It takes place on a road with a pedestrian and a stationary vehicle on a straight road. The learning goal is not to learn to handle the road as in Task 1, but to learn to maintain a safe speed in relation to the irregular crossing behaviors of a pedestrian. In this task, a pedestrian is added to the scene at various locations, both on and at the side of the road. The pedestrian walks along a random path to cross at an unexpected moment with various speeds and stops. In addition, a stationary car is added to the scene that may occlude the pedestrian (Figure 6). The goal of the vehicle is to travel along the road while regulating the speed to ensure pedestrian safety.

#### 3.4.1. Reward function

In this application the learning agent receives a positive reward as long as the controlled vehicle is driving at the preferred speed limit when it is within a safe distance of the pedestrian and also if the agent travels at a slower speed than the pedestrian while the agent is in a predefined “red” zone close to the pedestrian. The agent gets a negative reward if it speeds up in the red zone or travels too slowly in a safe zone. Finally, hitting the pedestrian immediately results in maximal punishment and ends the episode.

#### 3.4.2. Road generation

The road generation mechanism, in this task, modulates the start and end points of the pedestrian, at which point to cross the road to which point on the other side, the speed of the pedestrian. This, together with a trained vehicle, could result in a number of possible scenarios. For example, an episode may begin with a pedestrian standing mid-way down the road far from the starting point of the car. In this case, the car could start approaching the pedestrian and then start decelerating when it comes closer to the pedestrian. It may have to stop completely and wait until the pedestrian has left the road before it can increase the speed again and complete the road. In another scenario, the pedestrian might initially stand near the stationary car on the opposite side of the road (see Figure 6) and then cross the road to the other side at a random moment. In this scenario, the pedestrian may not be visible immediately or may initially be visible and then be occluded by the stationary car before emerging again on the road. As the order of difficulty is subjective to the designer, the generation mechanism is generally unstructured, yet the scenarios are grouped and ordered by similarities and a variety of motion movements in the road. The road generation produced a total of 1,000 different pedestrian crossing scenarios for the training. As this task deals with a higher level of control, the agent network encounters very similar experiences in the sequence of time steps; therefore, a replay buffer is used to overcome these similarities. The replay buffer setup is similar to the one used in the first task.

#### 3.4.3. Training phase

During training the network parameter of the learning agent was similar to that described in Table 1. However, the input includes information about the distance from the pedestrian, except when the pedestrian is occluded. The output is the action to regulate the speed of the vehicle by accelerating or decelerating. In the training phase, the agent interacts with different scenarios of the pedestrian to learn the safe speed that corresponds to the pedestrian movement. The agent needs to learn and finish the road successfully to move to the next until it finishes the 1,000 roads.

## 4. Results

### 4.1. Lane keeping task

**Figure 7:**
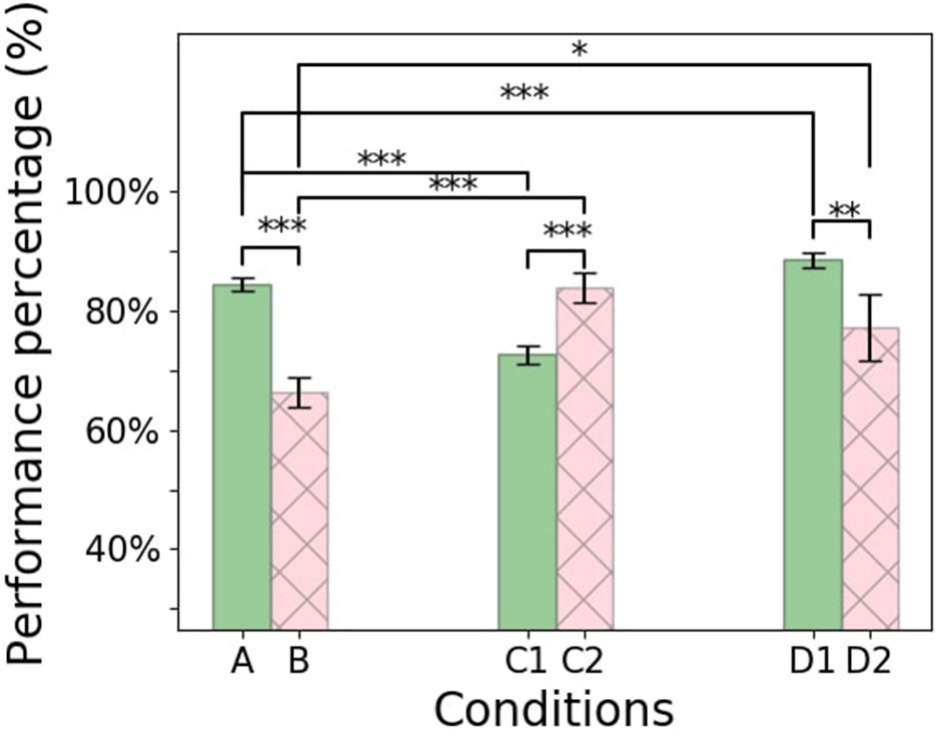
Learning agent performance measured by the percentage of the average total rewards out of the theoretical total reward at the testing phase for the six experimental conditions. Error bar indicate 95% confidence intervals. The figure shows the statistical significance between conditions, in which * for *P* ≤ 0.05, ** for *P* ≤ 0.01, and *** for *P* ≤ 0.001.

**Figure 8:**
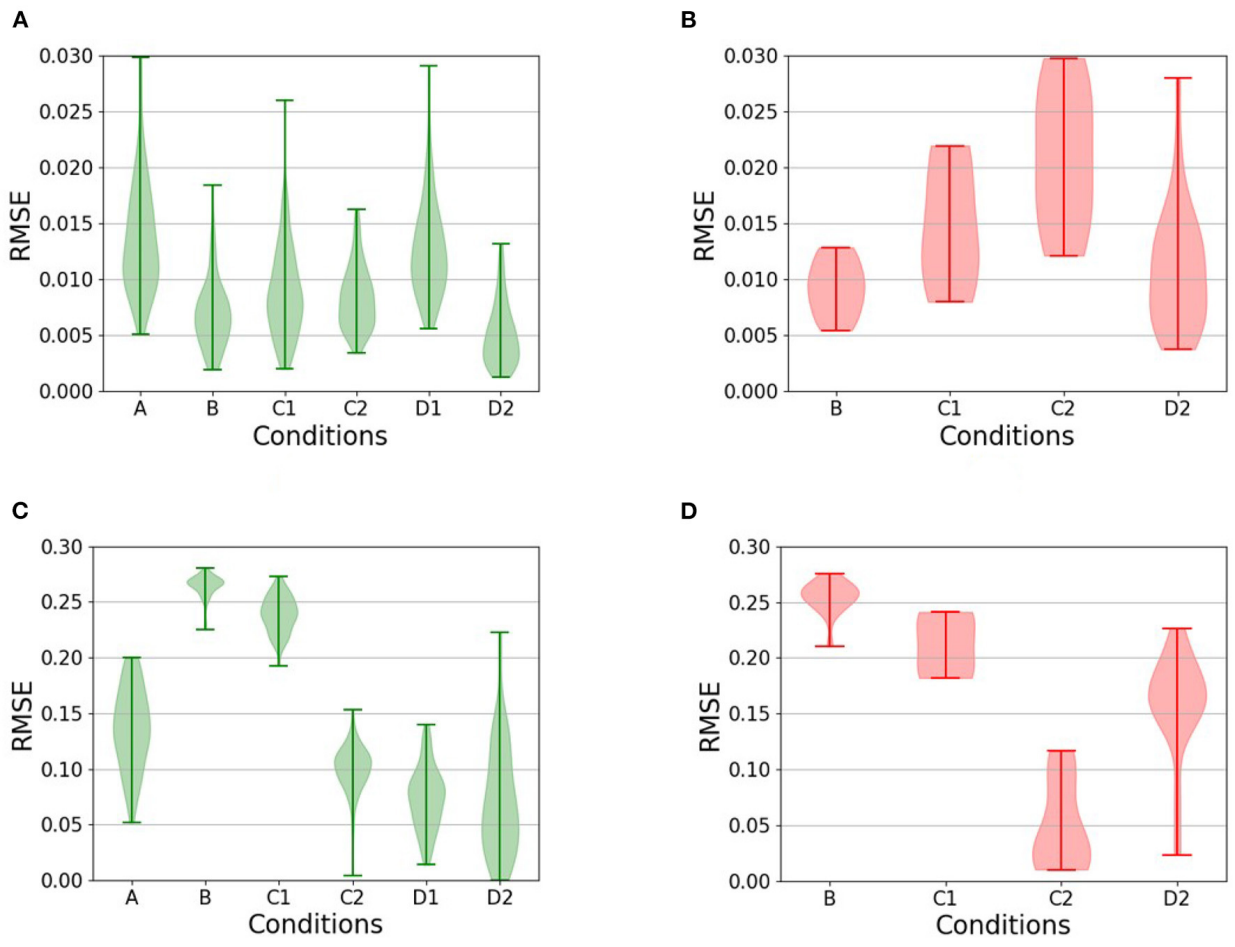
Violin plots comparing the Root Mean Square Error (RMSE) during testing for the relevant conditions (conditions for which there are no unsuccessful runs are omitted from the plot in question). **(A, B)** The RMSE of the heading with respect to the road heading, for **(A)** successful and **(B)** unsuccessful completions of the test roads. **(C, D)** The RMSE of the distance from the center of the road, also for **(C)** successful and **(D)** unsuccessful runs. Comparisons between successful and unsuccessful runs show, with the notable exception of [C2], that failure is typically a result of a failure to remain near the center of the lane coupled with an inability to maintain heading.

To evaluate the impact of synthetic data generation on learning, all agents are tested on 100 new randomly generated roads. The mechanism and structure of generation for the testing phase are similar to Condition [C1] with the 40:60 ratio between the curvy segments and the straight segments. The agent relies on the trained network for the policy selection. A 10% error is injected in the testing (the exploration rate is set to ϵ = 0.1) to combat overfilling and hinder the test. The agent's performance is defined as the average total reward received on the 100 test roads. The reward function captures how well the vehicle drives based on remaining (1) as close as possible to the middle of the road and (2) aligned with the lane heading. The theoretical maximum mean total reward is 8,500 (if the agent scores the maximum reward of five at each of the 1,700 steps per episode at the entire 100 episodes). The overall performance is illustrated as the percentage of the theoretical maximum (Figure 7) and will be described in detail in this section. The Root Mean Square Errors (RMSE) for each condition are presented in Figure 8. A Nonferrous correction was applied to the multiple *t*-test comparisons.

#### 4.1.1. Control condition [A]

During the training phase, the vanilla RL agent exhibited rather low performance during most of the trials, as evidenced by the low reward scores. The mean total reward in each episode during training was 2,060 (24% of the theoretical maximum), and the agent finished training after 640 episodes and 410,000 experiences.

During the testing phase, the agent completed all the 100 testing roads successfully, with a mean total reward of 6,750 corresponding to 79% of the theoretical maximum.

#### 4.1.2. Replay buffer only [B]

Training ended after 216 episodes and 245,000 experiences. The total rewards for each episode increased throughout the training and resulted in an average reward of 4,560 (54% of the theoretical maximum).

During the testing phase, the use of the replay buffer resulted in a mean total reward of 5,300 corresponding to 62% of the theoretical maximum, and the agent was unable to complete some of the roads successfully. The performance is significantly lower than the control condition [A] (*t*-test: *p* < 0.0001). It can also be observed in Figures 8A, C that the agent tends not to achieve a minimal RMSE with respect to the distance to the center of the lane. This suggests that when the agent is also unable to maintain headings (compare Figure 8A with Figure 8B), it has an increased likelihood of leaving the lane altogether.

#### 4.1.3. Unstructured synthetic data generation [C1]

During training, the agent showed unstable performance, performing well on some roads and poorly on others. The average total reward was around 5,700 (67%), and the duration of the training was similar to condition [A].

In the testing phase, the agent collected a mean total reward of 5,800 corresponding to 68% of the theoretical maximum. This is significantly worse than the control condition [A] (*t*-test *p* < 0.0001). It can again be observed in Figure 8 that the driving agents here do not minimize the RMSE of their distance to the center of the lane, resulting in a higher probability of leaving the road, when unable to maintain the headings.

#### 4.1.4. Unstructured synthetic data generation with replay buffer [C2]

The training ended in 200 episodes with 230,000 experiences. Most of the training time, 90 out of the 200 episodes, was spent on the first road. Once the agent had successfully learned to complete this, it was able to drive on the remaining roads with fewer failures.

The addition of the replay buffer compared to condition [C1] resulted in a significant improvement (*t*-test *p* < 0.0001) with a mean total reward of 6,700 corresponding to 79% of the theoretical maximum and on par with the control condition [A] (*t*-test *p*>0.5). Interestingly, this is the only condition in which the driving agents remained largely close to the center of the lane during unsuccessful runs. Instead, failure results entirely from the inability to maintain an appropriate heading with respect to the direction of the road, as can be seen from the heading RMSE (Figure 8B).

#### 4.1.5. Structured synthetic data generation [D1]

The training time of this condition was similar to that of condition [A]. The average total reward was 5,900 which is 70% of the theoretical maximum.

However, in the testing phase, this condition achieved the highest scores of all, with a mean total reward of 7,000, which corresponds to 82% of the theoretical maximum. The improvement was significant (*t*-test *p* < 0.05) compared to all other conditions (see Figure 7).

#### 4.1.6. Structured synthetic data generation with replay buffer [D2]

The training ended in 230 episodes and 245,000 experiences. The driving agent struggled mainly in the first 50 episodes. The total rewards gradually increased during training, but performance decreased in some episodes, likely due to increased complexity on the road at those moments.

In the testing phase, the addition of a replay buffer to condition [D1] resulted in a mean total reward of 6,100 corresponding to 72% of the theoretical maximum. This is significantly worse than condition [D1], which had an identical setup, except for not using a replay buffer (*t*-test *p* < 0.005). As can be seen from the RMSE of the distance from the center of the road (Figure 8D), the main reason for failure in this condition was, once more, the inability to remain in the middle of the road.

### 4.2. Pedestrian avoidance task results

**Figure 9:**
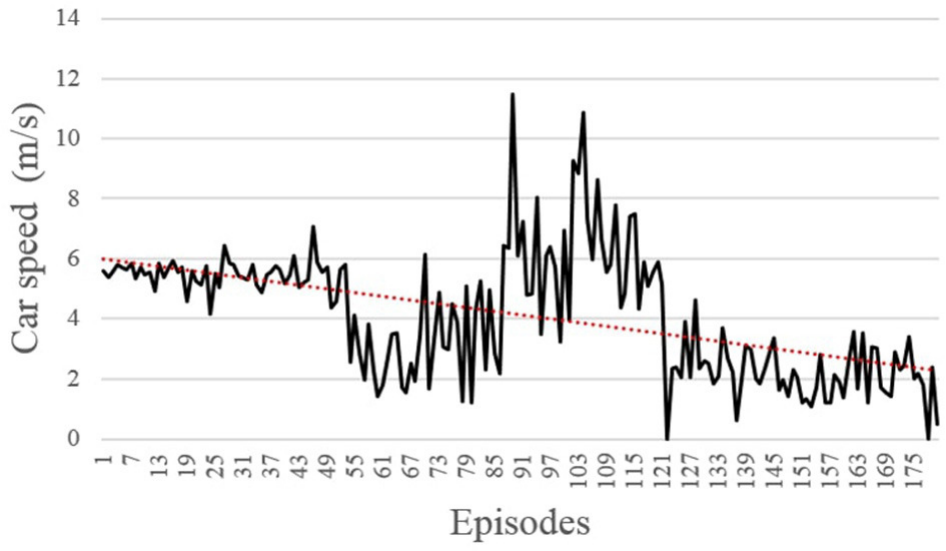
The speed at the collision with the pedestrian during training for collision cases.

The agent learns to successfully avoid the pedestrian through speed control. Overall, the training was relatively fast and in total around 180 pedestrian collisions were observed among the 1,000 training episodes (Figure 9). Despite these collisions, the average speed of the car at the time of collision was gradually decreasing, as expected.

The testing phase consisted of 100 pedestrian scenarios and no collisions were recorded in any of these scenarios. The results show that the speed control agent successfully drives at the optimal speed when it is far from the pedestrian and slows down when it is about to reach the pedestrian.

**Figure 10:**
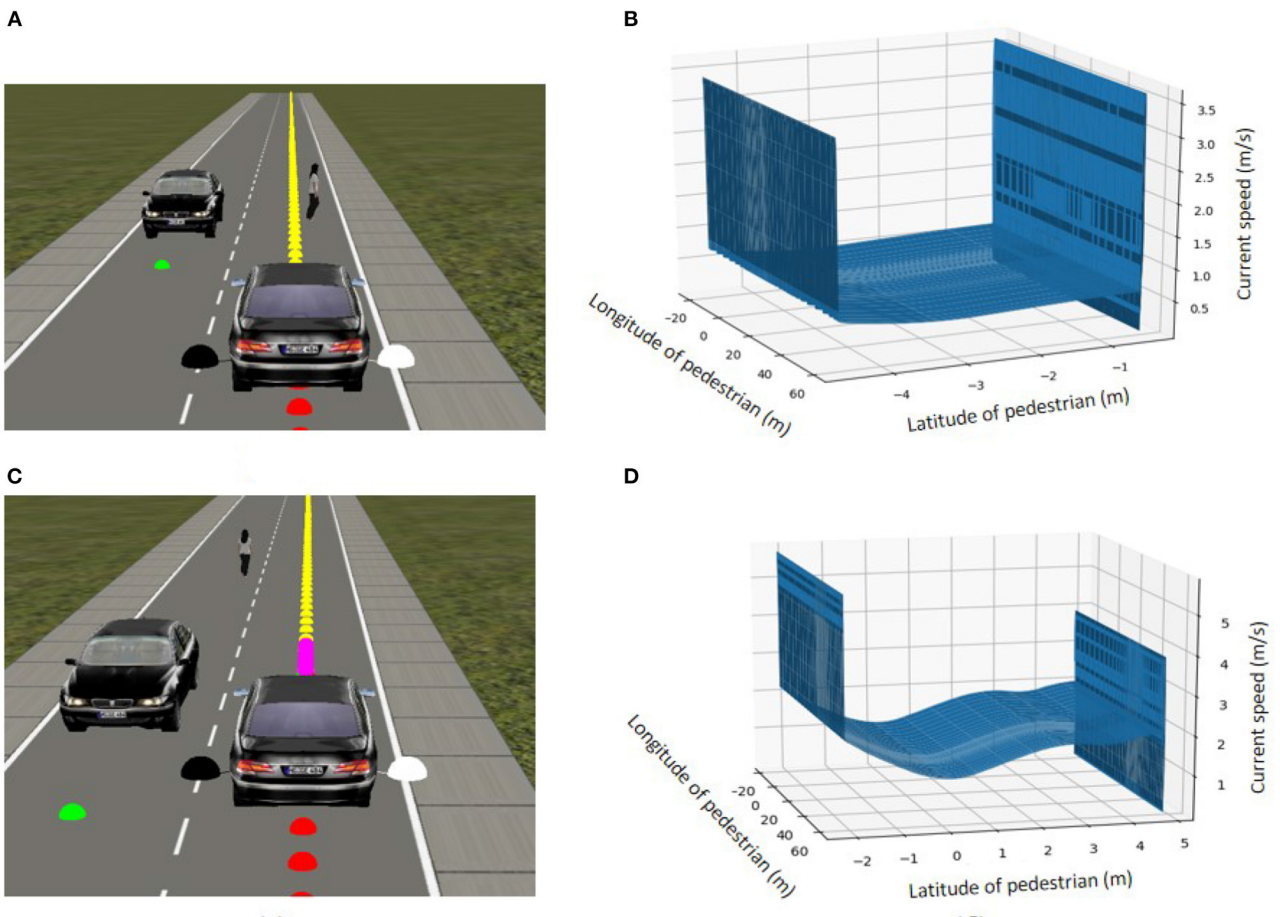
Two examples from the 100 pedestrian occlusion test scenarios. **(A)** A pedestrian stops in front of the car before continuing to cross the road. **(B)** Speed of the vehicle based on the position of the pedestrian with respect to the vehicle (positive latitude indicates distance to the left of the car, negative latitude distance to the right. A latitude of 0 means that the pedestrian is directly in front of the car). The vehicle stops whenever the participant is in front of it. **(C)** A pedestrian crossing the road diagonally from behind the stationary vehicle. **(D)** Speed of the vehicle based on the position of the pedestrian as above.

Since the vehicle performs well and is in line with expectations, we omit a lengthy elaboration of this basic result. In terms of illustrations, we present two of the 100 test cases. Figure 10A shows the scenario of a pedestrian standing still in the middle of the road. The vehicle was able to stop for the pedestrian until it crossed and then continued driving. The speed adjustment is in relation to the position of the pedestrian with respect to the vehicle, as shown in Figure 10B. When the pedestrian is in the vehicle's way, the speed control agent stops until this is no longer the case. Another test scenario included an occlusion, since the pedestrian crosses the road diagonally from behind a stationary vehicle (Figure 10C). The results in Figure 10D show that the vehicle slows down as long as the pedestrian is on the road. The vehicle accelerates only when the pedestrian safely reaches the sidewalk. Overall, it appears that the vehicle is able to learn multiple pedestrian behaviors and adjust its behavior to the environmental context, and drive at a safe speed in relation to the location of the pedestrian.

## 5. Discussion and conclusion

Self-driving cars have been an ideal test case for many practical applications of machine learning and cognitive systems in recent times, not least because they promise an autonomous agent solving non-trivial problems in a real environment (Mahmoud et al., 2022). However, many interesting challenges remain unsolved, including how vehicles can learn autonomously and robustly to drive safely even in rare events or situations that the system developer does not anticipate (Da Lio et al., 2017).

In this paper, we investigated ways to synthetically generate additional training data, both by creating novel scenarios for the vehicle (which can be learned either in a curriculum-like manner, that is, ordered by increasing difficulty, or in a random order) and by reusing existing scenarios using a replay buffer. This combination has been explored previously in an article (Fang et al., 2019); however, in that work, rather than retrieving stochastic experiences from the stored buffer, curriculum learning was used to gradually learn from these experiences. Here, we use curriculum learning at the scenario generation level so that the vehicle learns easier driving scenarios before turning to more sophisticated ones.

Our results showed that curriculum learning is a scenario generation method that can improve the learning process, but should not be taken for granted. Different combinations of episode generation mechanisms and usage of replay buffer had positive and negative effects on overall performance. In particular, the replay buffer, which is a default mechanism in RL, did not always show a positive result. It had a detrimental effect when used on the vanilla RL agent and on the agent using curriculum learning to structure training episodes. Although the replay buffer did improve the performance of unstructured learning using new training scenarios, the performance did not reach the best performing agent (which used only curriculum learning).

This detrimental effect could be due to the probability of sampling the experiences from the buffer. At each step, a mini batch of 36 is sampled from the 1,000 buffer experiences, which means that each sample is selected with a probability of 0.036 at each time step. In addition to the low probability of being selected, old experiences are also removed from the buffer and replaced by new experiences. This increases the likelihood that many potentially useful examples will never be seen by the network. This was also supported by additional tests (not reported in detail here) of different buffer sizes (100, 1,000, 100,000, and 1,000,000), which showed that larger buffers, i.e., lower likelihood of being selected, showed even worse performance. On the contrary, without the use of a replay buffer, each experience is ensured to contribute to network learning. This shows that the structure of generating the episode contributes to the learning more than the replay buffer technique, which may not always be beneficial for learning.

More generally, although some studies have found the technique to be useful for various tasks (Mnih et al., 2013; Lillicrap et al., 2015), the added value of replay buffers has also been questioned before. Zhang and Sutton (2017), for example, evaluated different experience replay mechanisms and found them to have a detrimental effect, noting, in particular, that “... [T]he idea of experience replay itself is heavily flawed. So future effort should focus on developing a new principled algorithm to fully replace experience replay” (Zhang and Sutton, 2017, p. 7).

Using GANs, as discussed earlier is a different way of generating new scenarios (not just individual experiences) (Ha and Schmidhuber, 2018). Although this method has gained a lot of attention for producing training data, it produces stochastic imagery data that may be unrealistic or not as helpful as it could be for the agent. Here, we investigated the added value of curriculum learning as yet another way of generating new, episodic, experiences from which the vehicle could learn. This approach differs from the GAN approach not just because of the structure of the training in terms of increasing difficulty, but also because of the use of a physics simulator to create actual novel scenarios as opposed to variations on sensorimotor imagery based on past experiences. The need for diverse and inclusive scenarios was emphasized by Codevilla et al. (2019), who trained a model on different sizes of collected driving data sets (2, 10, 50, and 100) hours of driving. They concluded that training in data sets larger than 10 h of driving does not ensure higher performance. It may even lead to negative effects when the data set does not include enough variety for rare scenarios to occur.

In this paper, different conditions were used to examine the benefit of generating synthetic data for generation. These conditions were used to compare the curriculum learning generation with other methods in the literature while using the same evaluation metric for comparison. Condition [A] which uses a single road with no replay buffer serves as the control condition. It represents the traditional usage of RL with a single environment in which the designer aims to cover the different scenarios in this training environment. Condition [B] represents the introduction of a replay buffer that was proposed to speed up the training. Although the literature has focused on the benefit of speeding up the training (Mnih et al., 2013; Lillicrap et al., 2015), less work has highlighted its drawback of degrading the training performance (Zhang and Sutton, 2017). Conditions [C1] and [C2] represent the promotion of machine learning to randomize the data to ensure variation and avoid overfilling. This shows similarities to the approach of creating random scenarios using neural networks such as GANs, but with less control from the designer. Our study showed that randomizing the episodes [as in condition (C1) would result in the agent encountering difficult scenarios at the beginning of the training which deteriorates the performance]. However, combining this with a replay buffer compensates for the difficulty of learning. Conditions [D1] and [D2] use curriculum learning, which is the main topic of study of this paper. Compared to the previous methods, curriculum learning showed an advantage when used without the replay buffer compared to the condition with the replay buffer.

Narvekar et al. (2022) proposed a taxonomy of curriculum learning for RL. The classification includes different factors of what the generation features focus on. The factors include the method of transferring the learning (e.g., re-shaping the reward function or the policy), the degree of designer involvement in the creation of the scenarios (e.g., GANs vs. the technique described in this paper), and the application area (e.g., grid world, physical simulation, or real-world deployment). In our study, the factors were selected to examine the use of curriculum learning for an application as self-driving cars.

The (re-)enacting of hypothetical situations such as it is achieved by the kind of episodic simulations used in this work has also received some attention in cognitive systems research in the past (see, for example, Tani and Nolfi, 1999; Ziemke et al., 2005; Hoffmann, 2007); however, the focus has typically been on simple neural networks driving simplistic agents (such as a simulated Kheda robot) in very simple environments (such as wall-following environments). Among others, implementing a more credibly biologically inspired approach requires such simulations to be more flexible with respect to their content (Revonsuo, 2000; Svensson et al., 2013). Using curriculum learning to structure such episodic situations can be a candidate way to achieve this.

To reiterate the main intention of this paper, our purpose was not to establish a new state-of-the-art in solving tasks such as lane keeping or pedestrian avoidance, but to explore the added value of generating additional synthetic training data, either based on past experience (using a replay buffer approach) or by generating novel scenarios. When generating novel scenarios, we hypothesized that it might be desirable to structure them in order of difficulty, and this is indeed what we found. At the same time, we noted that it may not always be possible to identify a clear difficulty metric and used the pedestrian avoidance scenario as an example. Although we initially intended to explore alternative ways of providing structure to the training scenarios in such a case, we found that the vanilla learning agent managed to perfectly solve this task without any further additions, which rendered that question obsolete. In a sense, the pedestrian detection task may seem simpler because it requires the manipulation of one degree of freedom (longitudinal control) compared to lane keeping, which requires manipulating two (adding latitudinal control). This is not a straightforward conclusion since the pedestrian detection task also takes place in a more dynamic environment, involving other agents, resulting in an increase in complexity in that sense.

The overall take-home message of this study is therefore that we do indeed find curriculum learning to be useful but that significant questions remain on how to structure such a curriculum precisely. A random scenario order of difficulty in the lane keeping task training turned out to be detrimental, demonstrating that there is a need for some difficulty metric to define the structure. Such a metric might not exist in all cases, in which case it is possibly better to avoid curriculum learning. In some cases at least, that does not have to be a disadvantage, as our second use case showed.

There remain, of course, many aspects of this work that can be improved in future research. The relative simplicity of the two tasks (and thus the obvious need to confirm the results in more complex scenarios) aside, even though we avoided hard-coding specific training and test scenarios, the exact structures used for road generation remained pre-defined. Future work should investigate agents' models that can be used to automatically determine a suitable sequence of episodes to train on, possibly online during the training phase. There are also interesting avenues that would involve incorporating progressive learning (e.g., Berseth et al., 2018). Combining both may lead to significant improvements in learning performance and advanced abilities to learn multiple driving skills.

## Data availability statement

The raw data supporting the conclusions of this article will be made available by the authors, without undue reservation.

## Author contributions

SM, ST, and HS: conceptualization. SM, EB, HS, and ST: methodology, investigation, and writing—review and editing. SM: software, validation, and data curation. SM and ST: formal analysis and visualization. ST and HS: resources, project administration, and funding acquisition. SM and HS: writing—original draft. ST, HS, and EB: supervision. All authors contributed to the article and approved the submitted version.

## Footnotes

^1^ https://opends.dfki.de/
